# Bilayer TiO_2_/Mo-BiVO_4_ Photoelectrocatalysts for Ibuprofen Degradation

**DOI:** 10.3390/ma18020344

**Published:** 2025-01-14

**Authors:** Martha Pylarinou, Elias Sakellis, Spiros Gardelis, Vassilis Psycharis, Marios G. Kostakis, Nikolaos S. Thomaidis, Vlassis Likodimos

**Affiliations:** 1 Section of Condensed Matter Physics, Department of Physics, National and Kapodistrian University of Athens, University Campus, 15784 Athens, Greece; pylarin@phys.uoa.gr (M.P.); e_sakel@phys.uoa.gr (E.S.); sgardelis@phys.uoa.gr (S.G.); 2 Institute of Nanoscience and Nanotechnology, National Center for Scientific Research “Demokritos”, Agia Paraskevi, 15341 Athens, Greece; v.psycharis@inn.demokritos.gr; 3 Laboratory of Analytical Chemistry, Department of Chemistry, National and Kapodistrian University of Athens, University Campus, 15771 Athens, Greece; makostak@chem.uoa.gr (M.G.K.); ntho@chem.uoa.gr (N.S.T.)

**Keywords:** BiVO_4_, TiO_2_, photocatalytic films, heterojunctions, photoelectrocatalysis, pharmaceuticals, ibuprofen

## Abstract

Heterojunction formation between BiVO_4_ nanomaterials and benchmark semiconductor photocatalysts has been keenly pursued as a promising approach to improve charge transport and charge separation via interfacial electron transfer for the photoelectrocatalytic degradation of recalcitrant pharmaceutical pollutants. In this work, a heterostructured TiO_2_/Mo-BiVO_4_ bilayer photoanode was fabricated by the deposition of a mesoporous TiO_2_ overlayer using the benchmark P25 titania catalyst on top of Mo-doped BiVO_4_ inverse opal films as the supporting layer, which intrinsically absorbs visible light below 490 nm, while offering improved charge transport. A porous P25/Mo-BiVO_4_ bilayer structure was produced from the densification of the inverse opal underlayer after post-thermal annealing, which was evaluated on photocurrent generation in aqueous electrolyte and the photoelectrocatalytic degradation of the refractory anti-inflammatory drug ibuprofen under back-side illumination by visible and UV–Vis light. Significantly enhanced photoelectrochemical performance on both photocurrent density and pharmaceutical degradation was achieved for the bilayer structure with respect to the additive effect of the constituent layers, which was related to the improved light harvesting arising from the backscattering by the mesoporous TiO_2_ layer in combination with the favorable charge transfer at the TiO_2_/Mo-BiVO_4_ interface.

## 1. Introduction

Nanostructured bismuth vanadate (BiVO_4_) has been established as a benchmark n-type semiconductor photoanode because of its exceptional performance in photoelectrochemical (PEC) water splitting, which originates from its favorable physical and chemical properties, including suitable valence band edge potential and visible light activated band gap, accompanied by environmental inertness, earth abundance, and relatively low cost as well as its long-term stability in neutral pH regions compared to other metal oxide PEC materials [1,2,3]. Major research efforts have been accordingly devoted to elaborate and optimize synthetic methods together with surface, bulk, and interface engineering of BiVO_4_ in order to alleviate persistent electron-hole recombination losses arising from the poor electron transfer kinetics that is the major drawback of BiVO_4_ photoelectrodes [4,5,6]. Despite the marked progress in the improvement of photoelectrochemical performance [7], application of BiVO_4_ photocatalysts in water purification has lagged behind as the relatively narrow band gap (2.4–2.6 eV) of the photocatalytically most active, monoclinic scheelite (*ms*) BiVO_4_ phase [8] may impede simultaneous generation of highly reactive oxygen species such as hydroxyl radicals (^•^OH) and superoxide radical anions (O^2−•^), which underlie the non-selective photocatalytic degradation of organic contaminants in the absence of sacrificial scavengers [9,10]. Recently, though, judicious compositional and morphology modifications have rendered BiVO_4_ an emerging photocatalyst in organics degradation under visible light, applied mostly in the photocatalytic removal of dye pollutants [11,12] and to a lesser extent on colorless water contaminants such as antibiotics and bacteria [13,14,15,16,17]. Advanced application of nanostructured BiVO_4_ photocatalysts has been further pursued on the photoelectrocatalytic degradation of refractory emerging contaminants such as pharmaceuticals [18], where simultaneous occurrence of both reduction and oxidation processes is not thermodynamically a prerequisite as the counter electrode can assist one of the redox reactions [19]. Metal doping of BiVO_4_ has been the most competent modification to enhance its poor charge transport/separation efficiency for PEC water splitting with Mo^6+^ and W^6+^ that replace lattice V^5+^ ions being the most efficient dopants [2,3]. Specifically, it has been established that the introduction of Mo-dopants in BiVO_4_ at low levels, which act as shallow donors, leads to the increase in donor density and n-type conductivity along with electron mobility and electron diffusion length by lowering the small polaron hopping barrier [20]. Heterojunction formation between pristine or doped BiVO_4_ nanomaterials with other semiconductor photocatalysts [21,22,23,24,25] and metal nanoparticles (NPs) [26,27,28] has accordingly attracted particular interest in order to improve charge transport and charge separation via interfacial electron transfer for the photoelectrocatalytic degradation of recalcitrant pharmaceuticals.

In addition to compositional engineering, tailoring the morphology of BiVO_4_ in the form of nanoporous photoanodes has been keenly pursued in order to enhance light harvesting and assist charge separation for photocatalytic applications [29,30,31,32]. Among different morphologies, nanocomposite photonic crystals (PCs) have been attracting attention for advanced photoelectric and absorption devices [33,34,35]. Inverse opals have been the most common three-dimensionally ordered structure, which, besides the selective enhancement of light harvesting via slow photons, offers a macroporous interconnected network that facilitates molecular transport and adsorption during photocatalytic reactions [36]. Nanoparticulate BiVO_4_ inverse opals have been successfully applied as visible light-activated photocatalysts in powder form [37,38], while Mo-doped BiVO_4_ inverse opal photoelectrodes have shown excellent performance in photoelectrochemical water splitting [39], especially after surface decoration by Au [40] and Ag [41] plasmonic NPs. Recently, markedly improved activity was reported for Mo-BiVO_4_/Ca-BiVO_4_ homojunctions [42] and (Ag, Au) plasmonic-modified Mo-BiVO_4_ inverse opal films [43] in the visible light photoelectrocatalytic degradation of pharmaceutical emerging contaminants. Heterostructuring of BiVO_4_ with benchmark TiO_2_ photocatalysts has been the subject of particular interest, where post-deposition of BiVO_4_ NPs has been commonly employed for the visible light sensitization of TiO_2_ inverse opals that were applied in the photocatalytic degradation of dye pollutants [44,45]. Likewise, BiVO_4_ NP decoration has been exploited for the improvement of water splitting over WO_3_ inverse opal photoanodes [46] as well as a nanocrystalline coating layer on top of WO_3_ [47] and TiO_2_ [48] inverse opal photoelectrodes leading to high photocurrent densities. Recently, BiVO_4_ NPs deposition on single [49] and bilayer [50] TiO_2_ inverse opal films resulted in greatly enhanced photocatalytic degradation rates of rhodamine B dye upon slow photon tuning, while significant enhancement of water oxidation was reported for a thin Mo-BiVO_4_ photoanode when coated with a TiO_2_ inverse opal layer with suitable photonic band gap with respect to the BiVO_4_ absorption edge [51].

In this work, heterostructured TiO_2_/Mo-BiVO_4_ films are demonstrated as efficient photoanodes for the photoelectrocatalytic degradation of recalcitrant pharmaceutical water pollutants. Bilayer photoelectrodes were fabricated by the deposition of a mesoporous TiO_2_ overlayer on top of Mo-BiVO_4_ inverse opal substrates, which intrinsically absorb in the visible range below 490 nm, while offering improved charge transport due to Mo-doping. Post-thermal annealing of the composite structure resulted in a porous P25/Mo-BiVO_4_ bilayer film after the collapse of the inverse opal bottom layer due to crystallite growth. Comparative PEC evaluation showed that the bilayer photoelectrodes present greatly improved performance with respect to the additive effect of the constituent layers in both photocurrent density and the photoelectrocatalytic degradation of ibuprofen (IBU), a non-steroidal anti-inflammatory drug under back-side illumination by visible and UV–Vis light. Optical and PEC measurements indicate that the rise of photoelectrocatalytic activity of the bilayer films is related to the enhanced light harvesting arising from backscattering by the mesoporous TiO_2_ layer in combination with the favorable electron transfer at the TiO_2_/Mo-BiVO_4_ interface.

## 2. Materials and Methods

### 2.1. Bilayer Fabrication

A Mo-doped BiVO_4_ inverse opal layer was initially fabricated using the liquid phase infiltration of a colloidal opal template of 340 nm diameter polystyrene (PS) spheres with the appropriate metal salt precursor and optimized composition. Specifically, the 3% Mo:V molar ratio and the templating sphere size were selected based on our recent work [42], where the Mo-doping levels and the template sphere size were varied in order to optimize the photoelectrocatalytic activity of Mo-BiVO_4_ on pharmaceutical degradation under visible light. The horizontal colloidal crystallization method was applied in order to rapidly deposit the PS template films without consuming large amounts of colloidal suspension as in conventional self-assembly methods [52]. Specifically, 100 μL of 1.25 wt% monodisperse PS sphere suspension (Microparticles GmbH, Berlin, Germany, standard deviation SD = 10 nm, 2.9% CV, in the form of 5% solids (*w*/*v*) colloidal dispersions in deionized water) was deposited on a horizontal fluorine-doped tin oxide (FTO, thickness 2.2 mm, surface resistivity 7 Ω/sq, Sigma Aldrich, St. Louis, MO, USA) conductive glass substrate, which was kept at 20 °C until the solvent evaporated. In that case, inward convective flow as the evaporation rate at the periphery is higher than that at the center results in the self-assembly of PS microspheres to the face-centered cubic (fcc) close-packed lattice. FTO substrates, prior to their use, have been cleaned by ultrasound sonication in Hellmanex™ III (Helma Analytics, Müllheim, Germany) and propanol-2, followed by dipping in water and drying in N_2_, while their hydrophilicity was enhanced by dipping in H_2_O:HNO_3_ (5:1). Then, liquid infiltration of the PS opal template was performed in a complex metal salt precursor based on ammonium vanadate (NH_4_VO_3_) (ACS, 99.0% min) and bismuth(III) nitrate pentahydrate, Bi(NO_3_)_3_·5H_2_O (99.999% trace metals basis) with the addition of ammonium molybdate tetrahydrate (NH_4_)_6_Mo_7_O_24_·4H_2_O (BioUltra, ≥99.0%) as source of molybdenum (Mo) doping [43]. Specifically, 0.5 mmol NH_4_VO_3_ was initially mixed with 9 mL of propanol-2 (HPLC, 99.8%) and 0.5 mL of nitric acid (puriss. p.a., 65%) under continuous stirring for 30 min at room temperature, followed by sonication and the addition of 0.5 mmol citric acid (anhydrous, ACS, 99.5+%) until dissolution. Mo-doping was implemented by substituting part of NH_4_VO_3_ with (NH_4_)_6_Mo_7_ O_24_∙4H_2_O at Mo:V molar ratio of 3%. Another solution was prepared by dissolving 0.5 mmol Bi(NO_3_)_3_·5H_2_O with 0.5 mL acetic acid (glacial, ACS, 99.7+%). The two solutions were mixed and stirred for 30 min to obtain the Mo-BiVO_4_ metal salt precursor, wherein the PS opal films were dipped for 5 min, followed by drying for 1 h at 70 °C. The dip-coating process was repeated three times in order to ensure complete precursor infiltration to the opal interstices followed by post-calcination 400 °C in air to remove the PS template and crystallize the amorphous precursor in the Mo-BiVO_4_ inverse opal structure. Reference films of undoped BiVO_4_ PC were likewise deposited on FTO substrates without adding the Mo-salt in the precursor mixture. The PC films were labeled as Mo-BiVO_4_ PC and BiVO_4_ PC, respectively.

**Scheme 1:**
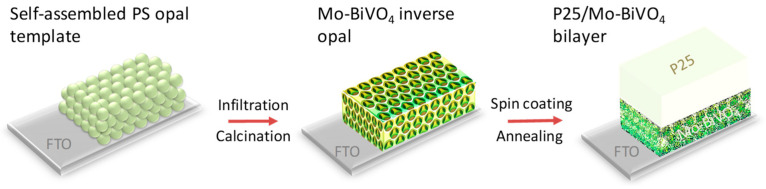
Fabrication process of P25/Mo-BiVO_4_ bilayer films.

The bilayer photoelectrode was then constructed by the deposition of a mesoporous nanocrystalline TiO_2_ layer [53] on the Mo-BiVO_4_ films, using Aeroxide P25 (Evonik, Essen, Germany) as benchmark TiO_2_ photocatalyst due to the mixed phase of anatase and rutile NPs and the concomitant interfacial charge transfer [54]. The deposition of the P25 paste on top of the Mo-BiVO_4_ PC films was performed by spin coating (Ossila, Sheffield, UK) at 400 rpm for 30 s followed by drying at 120 °C and post-annealing at 450 °C in air for 30 min with rate 5 °C/min, in order to remove organics, leading to P25/Mo-BiVO_4_ films (Scheme 1). In addition, Mo-BiVO_4_/P25 bilayer films with reversed deposition sequence i.e., a mesoporous P25 underlayer was first coated on the FTO substrate followed by the Mo-BiVO_4_ inverse opal deposition, were fabricated in order to determine the optimal bilayer structure. Nanocrystalline Mo-BiVO_4_ planar films and the corresponding bilayers were also deposited as reference films, applying the same procedure without the colloidal template.

### 2.2. Materials Characterization

The films’ morphology and phase composition were characterized using a scanning electron microscope (SEM) (FEI Quanta Inspect, Eindhoven, The Netherlands) coupled with an energy-dispersive X-ray (EDX) analyzer (EDAX, Mahwah, NJ, USA) and an FEI Talos F200i scanning transmission electron microscope (TEM) (Thermo Fisher Scientific Inc., Waltham, MA, USA) operating at 200 keV, equipped with a windowless energy-dispersive spectroscopy microanalyzer (6T/100 Bruker, Hamburg, Germany). The materials’ structural properties were investigated by X-ray powder diffraction (XRD) using a SmartLab Rigaku θ/θ Bragg–Brentano diffractometer (Rigaku, Tokyo, Japan), equipped with a pyrolytic graphite monochromator using Cu Kα radiation. The power conditions were set at 40 kV/35 mA and the aperture slits were set at 2/3°, 2/3°, 0.6°. The continuous step-scanning technique was used at steps of 0.03° with measuring time of 11 s/step and the recorded 2θ range was 15–55°. The films’ phase composition was further studied by micro-Raman spectroscopy using a confocal Raman microscope (LabRAM Soleil™, Horiba Scientific, Longjumeau, France) equipped with two automated built-in lasers emitting at 532 and 785 nm. The laser beam was focused on the films’ surface using a 100× (NA = 0.9) objective on a high-end microscope with a motorized high-precision X-Y-Z sample stage. The scattered light was filtered by edge Rayleigh rejection filters with cutoffs at 50 cm^−1^ and analyzed by a 330 mm focal length spectrometer, a 1800 gr/mm diffraction grating, and a high-sensitivity UV–Vis CCD detector (Horiba Scientific, Longjumeau, France). Diffuse and specular reflectance measurements were performed using a fiber-optic diffuse and a 15° specular reflectance accessory on a Cary60 UV–Vis spectrometer (Agilent, Santa Clara, CA, USA), respectively. A Halon reference and a UV-enhanced Al mirror were used for background determination.

PEC experiments were performed in a three-electrode configuration on a CS350 potentiostat/galvanostat (Corrtest Instruments, Wuhan, China) using the single and bilayer films on FTO substrates as working electrode, Ag/AgCl as reference, and a Pt foil as counter electrodes in 0.5 M NaHCO_3_ aqueous electrolyte. The PEC measurements were carried out under dark and back-side illumination using a 300 W Xe lamp as source of UV–Vis and visible light (400 nm cutoff filter, 100 mW/cm^2^). Linear sweep voltammetry was performed at a potential scan rate of 10 mV s^−1^. The applied potential vs. Ag/AgCl was converted to reversible hydrogen electrode (RHE) scale by using the following equation:

*E*_RHE_ = *E*_Ag/AgCl_ + 0.059 pH + *E*^0^_Ag/AgCl_, (1)

where *E*^0^_Ag/AgCl_ = 0.1976 V is the standard potential for Ag/AgCl at 25 °C. Electrochemical impedance spectroscopy (EIS) was performed at the open-circuit voltage, in the 10^4^–10^−2^ Hz frequency range with ac amplitude of 10 mV. Mott–Schottky measurements were carried out at 500 Hz and a scan rate of 10 mV/s at each potential. The flat band potential and donor densities for the PC photoelectrodes were determined from the corresponding Mott–Schottky plots, i.e., the variation of 1/*C*^2^ vs. applied potential, using the following relation:

(2) $$\frac{1}{C^{2}}=\frac{2}{eA^{2}\varepsilon \varepsilon_{0}N_{D}}\left(V-V_{fb}-\frac{kT}{e}\right),$$

where *C* is the space-charge capacitance, *e* is the elementary charge and *A* is the electrode area, *N*_D_ is the donor density, *ε* is the semiconductor permittivity and *ε*_0_ the vacuum permittivity, *V*_fb_ is the flat band potential, *T* is the absolute temperature, and *k* is Boltzman’s constant. Incident photon-to-current efficiency (IPCE) measurements were also performed in the same PEC cell in 0.5 M NaHCO_3_ aqueous electrolyte under back-side illumination at 1.23 V vs. RHE potential using a 1 kW Xe lamp in combination with an Oriel 77200 1/4 monochromator (Newport, Irvine, CA, USA) and a calibrated Si photodiode. IPCE was calculated according to the following relation:

(3) $$IPCE\left(\%\right)=\frac{1239.8\left[V\;nm\right]\times J\left[mA/{cm}^{2}\right]}{\lambda \left[nm\right]\times P\left[mW/{cm}^{2}\right]}\times 100$$

where J is the photocurrent density in $mA/{cm}^{2}$, λ is the wavelength of incident light in nm, and P is the monochromatic light power density $\left(mW/{cm}^{2}\right)$.

### 2.3. Photoelectrocatalytic Performance

Photoelectrocatalytic experiments were also carried out in three-electrode configuration under back-side illumination by a 300 W Xe lamp in combination with a 400 nm long-pass filter (100 mW/cm^2^) using 36 mL working solution of 0.1 M NaHCO_3_ supporting electrolyte under mild stirring, comprising 5 mg/L of IBU pollutant at pH = 4. Degradation kinetics was monitored by spectrophotometric detection for small aliquots periodically withdrawn from the reaction cell. The degradation tests were performed in triplicate, and standard errors were calculated for the mean kinetic constants. The IBU concentration was also monitored by high-performance liquid chromatography (HPLC) under UV–Vis light up to 5 h for the P25/Mo-BiVO_4_ bilayer photocatalyst using an Agilent 1260 Infinity II (Agilent, Santa Clara, CA, USA) HPLC-DAD equipped with a quaternary pump G7111B, multicolumn thermostat G7116A, vial sampler G7129A and DAD HS G7117C. Chromatographic separation was achieved with ZORBAX eclipse plus C8 column (150 mm × 4.6 mm, 5 μm), provided by Agilent. For the separation, the mobile phase consisted of Solution of Monobasic Potassium Phosphate in water and Tetrahydrofuran (56:44, *v*/*v*) adjusted in pH 2.0 with phosphoric acid. The column temperature was maintained at room temperature, the injection volume was 20 μL, and the flow rate 1 mL/min.

## 3. Results

### 3.1. Morphological, Structural, and Optical Properties

**Figure 1:**
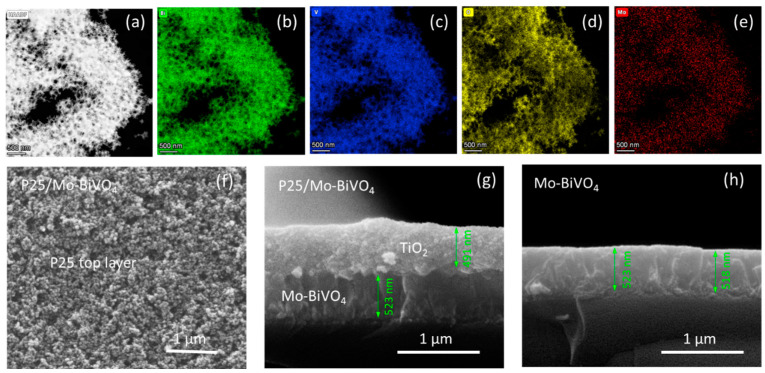
(**a**) TEM image of the Mo-BiVO_4_ PC underlayer and the corresponding (**b**) Bi, (**c**) V, (**d**) O, and (**e**) Mo EDX elemental maps. (**f**) Top view and (**g**) cross-section SEM image of the P25/Mo-BiVO_4_ bilayer structure. (**h**) Cross-section SEM image of the bottom Mo-BiVO_4_ layer.

TEM images of the Mo-BiVO_4_ PC supporting layer that was initially deposited on the FTO glass substrates indicate the formation of an ordered inverse opal structure with macropores of about 200 nm size (Figure 1a), whose chemical composition was confirmed by the corresponding EDX elemental maps of Bi, V, O, and Mo (Figure 1b–e). Subsequently, a mesoporous P25 TiO_2_ layer was deposited on top of the Mo-doped BiVO_4_ PC film (Figure 1f). Post-thermal treatment resulted in the formation of a porous bilayer structure comprising a nanocrystalline Mo-BiVO_4_ bottom layer produced from the collapse of the inverse opal structure after the second thermal annealing and the resulting crystallite growth and a mesoporous titania overlayer consisting of interconnected P25 aggregates, each layer being about 500 nm thick (Figure 1g,h).

**Figure 2:**
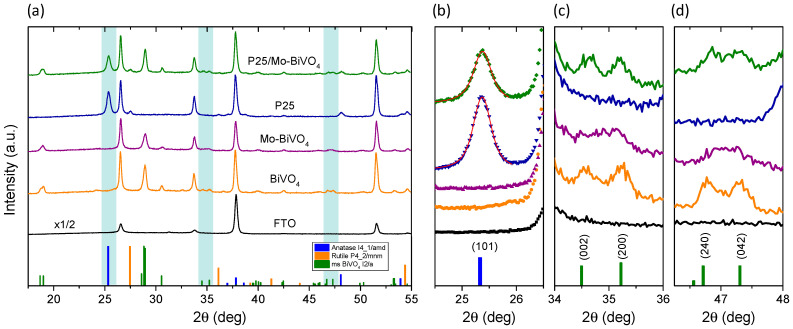
(**a**) XRD patterns of the P25/Mo-BiVO_4_ bilayer in comparison to the individual Mo-BiVO_4_ and P25 layer, the undoped BiVO_4_ PC film, and the FTO substrate. Shaded bands indicate the 2θ regions where the variations of the (**b**) main (101) diffraction peak of the anatase TiO_2_ phase of P25, and the splitting of the (**c**) (002)/(200) and (**d**) (240)/(042) diffraction peaks of the *ms* BiVO_4_ phase, are shown in detail. Red solid lines in (**b**) depict the best fits of the (101) peaks to Voight profiles with full widths at half-maximum of 0.40 and 0.41 deg for the P25 and P25/Mo-BiVO_4_ bilayer films.

XRD measurements on the single BiVO_4_ and Mo-BiVO_4_ films as well as the P25/Mo-BiVO_4_ bilayers (Figure 2a) confirmed that the films crystallize in the single *ms* phase (space group I2/a). This can be clearly evidenced by the characteristic splitting of the (002)/(200) (Figure 2c) and the (240)/(042) (Figure 2d) diffraction peaks. However, the observed peak splitting was appreciably reduced and less distinct for Mo-BiVO_4_.

This verifies that the introduction of Mo^6+^ dopants, which can substitute for V^5+^ cations in the BiVO_4_ lattice, moderate the monoclinic distortion of the *ms* phase towards the tetragonal scheelite (space group I4_1_/a) structure [55]. However, a reentrant increase in the splitting and a narrowing of the *ms* diffraction peaks were observed for the P25/Mo-BiVO_4_ bilayer films (Figure 2c,d), suggesting the growth of Mo-BiVO_4_ crystallites following the post-thermal annealing that was applied during the deposition of the nanocrystalline P25 titania top layer. The XRD pattern of the individual P25 films deposited on FTO showed the characteristic mixed phase of P25 nanocrystalline titania, consisting of a major anatase (space group *I*4_1_/*amd*) TiO_2_ phase along with secondary rutile (space group *P*4_2_/*mnm*) phase [49]. However, deposition of the P25 layer on the macroporous Mo-BiVO_4_ inverse opal substrate resulted in the slight broadening of the main (101) anatase peak (Figure 2b), which indicates the possibility for a modified interfacial layer between the TiO_2_ and Mo-BiVO_4_ NPs during the post-annealing treatment.

**Figure 3:**
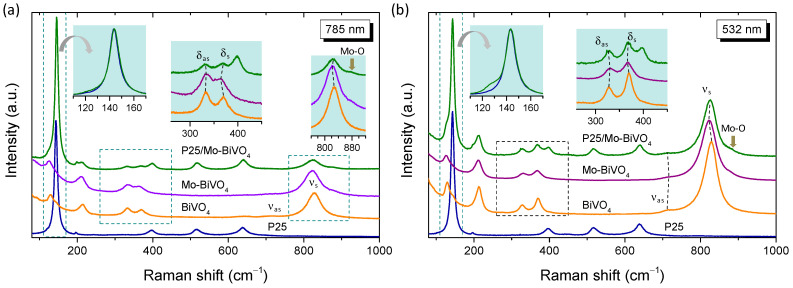
Raman spectra of the P25/Mo-BiVO_4_ bilayer in comparison to the single BiVO_4_, Mo-BiVO_4_ and P25 layers at (**a**) 785 and (**b**) 532 nm excitation wavelengths. Dashed boxes delineate the spectral regions that are shown in detail in the shaded insets. In the left inset for each laser excitation, the anatase E_g_ modes of the P25 and P25/Mo-BiVO_4_ films are compared after background removal at normalized intensity scale. Arrows indicate the weak shoulder that is associated with the Mo-O stretching vibration for the Mo-BiVO_4_ layers.

The phase composition of the individual layers and the bilayer structure was further examined by micro-Raman spectroscopy at 785 and 532 nm excitations (Figure 3a,b). The individual BiVO_4_ films exhibited all the characteristic Raman-active modes of the *ms* phase, without any evidence of polymeric residues or other BiVO_4_ polymorphic phases under both excitations. Notably, the strong symmetric (ν_s_) V-O stretching mode appeared at 828 cm^−1^, along with the much weaker antisymmetric (ν_as_) band (~712 cm^−1^), which was more clearly observed at 532 nm (2.33 eV). The latter energy approaches the band gap of BiVO_4_ (*vide infra*) leading to enhanced Raman scattering due to near-resonant excitation. The *ms* BiVO_4_ formation was confirmed by the observation of two distinct bands at 369 and 330 cm^−1^, corresponding to the symmetric (δ_s_) and antisymmetric (δ_as_) bending modes of the VO_4_ tetrahedra, as well as two intense external lattice modes at 213 and 129 cm^−1^ [42,56,57]. The effect of Mo-doping was evident through the shifts of the Raman modes compared to the pristine BiVO_4_ films. The ν_s_ and ν_as_ stretching modes, as well as the δ_s_ and δ_as_ bending ones, shifted closer together in Mo-BiVO_4_, indicating a reduction in the difference between the two V-O bond lengths and a decrease in the VO_4_ tetrahedral deformation of the *ms* phase after Mo-doping, in accordance with previous works [42,55,57]. In addition, a weak shoulder appeared around 880 cm^−1^ after Mo-doping, associated with the MoO_4_ stretching vibration [20,55]. A reentrant behavior of the Mo-BiVO_4_ Raman modes towards those of undoped BiVO_4_ was observed in the P25/Mo-BiVO_4_ bilayer, especially under near-resonant excitation at 532 nm, which enhanced the Mo-BiVO_4_ Raman intensity relative to the TiO_2_ top layer’s Raman modes (Figure 3b). This suggests that post-annealing during P25 layer deposition improved the crystallinity of the Mo-BiVO_4_ underlayer consistently with the XRD results. The individual P25 layers exhibited the characteristic Raman-active phonons of the major anatase TiO_2_ phase at approximately 144 (E_g_), 196 (E_g_), 397 (B_1g_), 517 (A_1g_ + B_1g_), and 638 cm^−1^ (E_g_), while the most intense Raman modes of the minor rutile TiO_2_ phase were traced around 445 and 610 cm^−1^ (not discernible in the intensity scale of Figure 3a,b) [58]. In this case, depositing the P25 layer on the Mo-BiVO_4_ substrate did not significantly affect the most intense, low-frequency anatase E_g_ mode, except for a slight broadening (left insets of Figure 3a,b), in contrast to the distinctive frequency shifts observed for the *ms* Raman modes due to the improved crystallinity of the Mo-BiVO_4_ substrate in the bilayer structure. This implies that any modification of the P25 layer in the bilayer structure is confined at the TiO_2_/Mo-BiVO_4_ interface, which contributes minimally to the Raman scattering volume.

**Figure 4:**
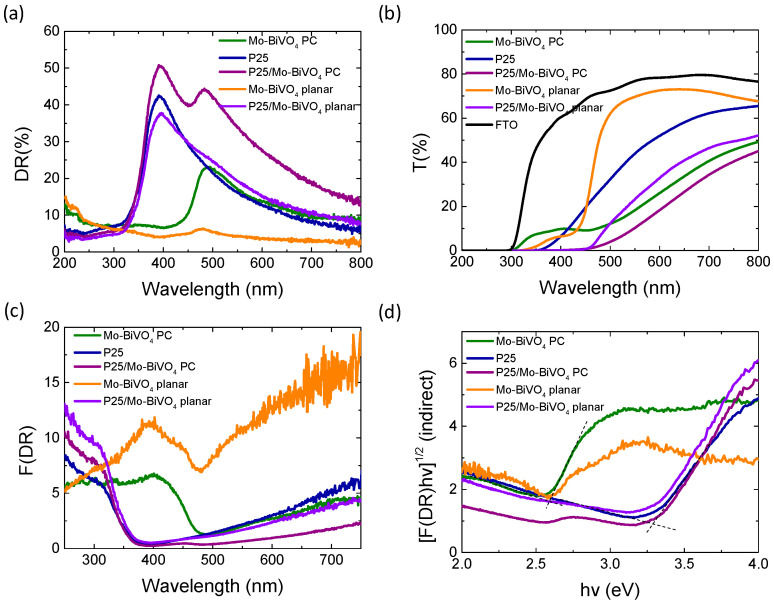
(**a**) Diffuse reflectance (DR%), (**b**) transmittance (T%) and the corresponding (**c**) Kubelka–Munk absorption, and (**d**) Tauc plots of the individual layers and the P25/Mo-BiVO_4_ bilayers.

Figure 4 shows the diffuse reflectance (DR%) and transmittance (T%) spectra of the P25/Mo-BiVO_4_ bilayer films compared to the individual layers. Both DR% and T% measurements showed the superposition of the absorption edges of the two constituent Mo-BiVO_4_ and TiO_2_ metal oxides, which were clearly resolved in the corresponding absorption spectra obtained from the Kubelka–Munk transforms of the DR% spectra. The derived Tauc plots for indirect band gaps indicated E_g_ values of 3.28 and 2.59 eV for the individual P25 and Mo-BiVO_4_ layers, respectively (Figure 4d). The corresponding E_g_ values for the P25/Mo-BiVO_4_ bilayer indicated a slight narrowing of E_g_ to 2.56 eV for Mo-BiVO_4_ that may be associated with the post-thermal annealing and the enhanced crystallinity in contrast to the identical value for the P25 upper layer. Comparison was also made with nanocrystalline Mo-BiVO_4_ planar films and the corresponding bilayers, which were deposited on FTO glass substrates using the same procedure without the colloidal template, in order to validate the role of the inverse opal structure in the films’ performance. These reference films showed qualitatively similar optical spectra though with considerably reduced DR% and enhanced T% arising from the markedly decreased DR% for the planar Mo-BiVO_4_ underlayer in comparison to the inverse opal supporting layer (Figure 4a), indicative of lower surface roughness and porosity.

### 3.2. PEC Properties

**Figure 5:**
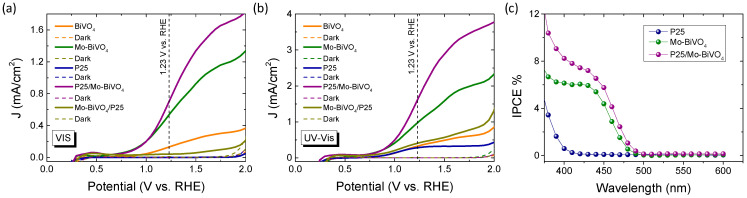
Photocurrent density-potential (J-V) curves for the P25/Mo-BiVO_4_ and Mo-BiVO_4_/P25 films in comparison to the individual layers and BiVO_4_ reference under (**a**) visible and (**b**) UV–Vis back-side illumination in 0.5 M NaHCO_3_ aqueous electrolyte. (**c**) IPCE spectra at 1.23 V vs. RHE potential for the P25/Mo-BiVO_4_ bilayer with respect to the P25 and Mo-BiVO_4_ constituent layers.

The PEC performance of the bilayer structures and the individual layers was subsequently investigated by photocurrent density-potential curves in 0.5 M NaHCO_3_ aqueous electrolyte under visible and UV–Vis back-side illumination (Figure 5a,b). The Mo-BiVO_4_ films presented increased photocurrent density compared to plain BiVO_4_, confirming the improved charge transport/separation efficiency arising from Mo-doping [2,3,20,42]. The P25/Mo-BiVO_4_ bilayer showed much higher photocurrent density than the reverse Mo-BiVO_4_/P25 bilayer structure under both illumination conditions, indicating greatly improved charge separation efficiency. On the other hand, charge transport and collection efficiency were greatly obstructed when the P25 layer was deposited directly on the FTO substrate, even under UV–Vis illumination, reflecting its poor PEC performance for water oxidation compared to Mo-BiVO_4_ photoanodes [2].

The photocurrent density of the Mo-BiVO_4_/P25 bilayer exceeded the corresponding sums of the individual Mo-BiVO_4_ and P25 layers by about 31% under both visible and UV–Vis light, indicating improved PEC performance by enhanced light capture and/or charge separation. The former was corroborated by IPCE measurements that showed appreciable enhancement for the P25/Mo-BiVO_4_ bilayer films, especially below the absorption edge (490 nm) of Mo-BiVO_4_ down to about 440 nm (Figure 5c). This suggests improved light harvesting by the Mo-BiVO_4_ layer from the backscattering of light that reached the mesoporous P25 film, which presents significant diffuse reflectance in this spectral range (Figure 4a).

**Figure 6:**
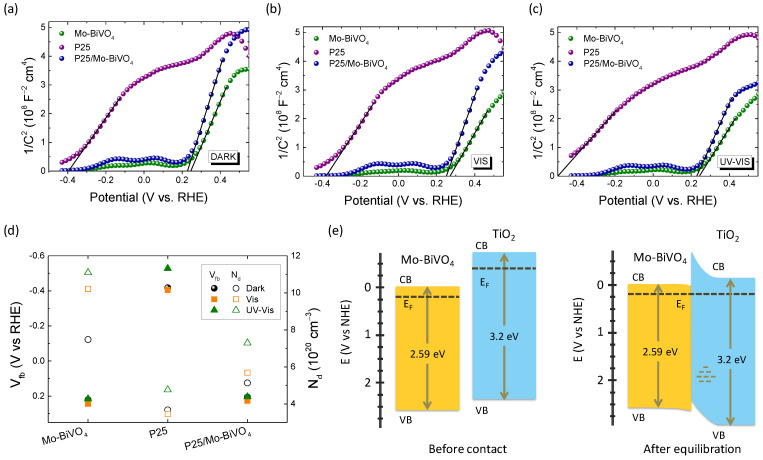
Mott–Schottky plots for the P25/Mo-BiVO_4_ bilayer in comparison to the Mo-BiVO_4_ and P25 individual films under (**a**) dark, (**b**) visible, and (**c**) UV–Vis light illumination in 0.5 M NaHCO_3_ aqueous electrolyte. (**d**) The derived values of *V*_fb_ and *N*_D_ and (**e**) tentative band alignment for the Mo-BiVO_4_/TiO_2_ heterojunction before and after Fermi level equilibration.

Charge transfer between the metal oxide layers was investigated by Mott–Schottky measurements, which were used for the estimation of the flat band potentials *V*_fb_ and donor densities *N*_D_ of the bilayer film with respect to its individual constituents (Figure 6). The Mo-BiVO_4_ and P25 films as well as the bilayer P25/Mo-BiVO_4_ presented positive slopes, indicative of n-type semiconducting behavior with considerably higher donor density *N*_D_ for the n-doped Mo-BiVO_4_ films [42]. The *V*_fb_ values for the Mo-BiVO_4_, P25 layers, and P25/Mo-BiVO_4_ bilayer that were determined from the intercept of the Mott–Schottky plots with the potential axis indicated significant offsets between the individual Mo-BiVO_4_ and P25 films. Specifically, the obtained *V*_fb_, which provide an estimate of the corresponding Fermi level positions, indicate considerable downshift of the energy band positions for Mo-BiVO_4_ with respect to that of the TiO_2_ top layer [59], leading to a staggered alignment between their conduction (CB) and valence (VB) bands before contact, as shown by the tentative band diagram of Figure 6e. When the two layers come into contact, their Fermi levels will align, close to that of Mo-BiVO_4_ whose *V*_fb_ is not appreciably shifted in the corresponding Mott–Schottky plots. The formation of a type I heterojunction (straddling band alignment) would be accordingly expected (Figure 6e), where both photogenerated electrons and holes will accumulate in the Mo-BiVO_4_ layer, which, however, is not favorable for the spatial charge separation between the two metal oxides. In that case, it is very likely that the formation of intermediate and/or defect states at the Mo-BiVO_4_/TiO_2_ interface could mediate hole transfer from BiVO_4_ to TiO_2_ despite the energy barrier between their VBs, as previously reported [3,60].

**Figure 7:**
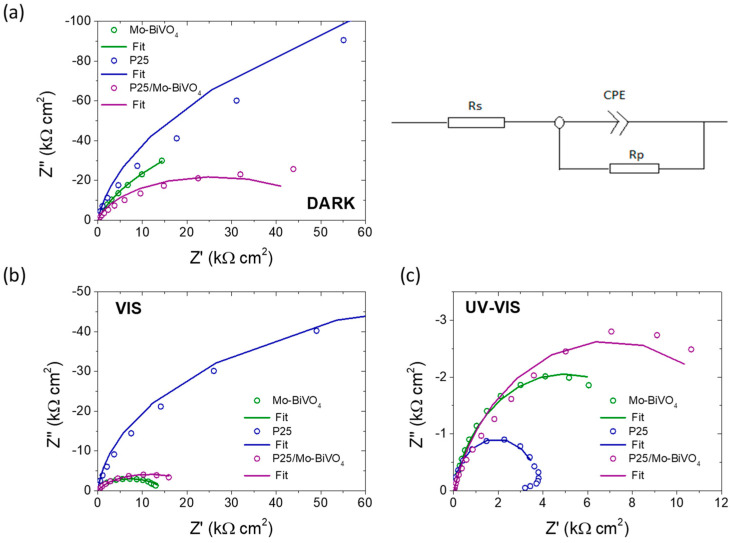
EIS Nyquist plots for the P25/Mo-BiVO_4_ bilayer in comparison to Mo-BiVO_4_ and P25 films under (**a**) dark, (**b**) visible, and (**c**) UV–Vis back-side illumination in 0.5 M NaHCO_3_ aqueous electrolyte. Black lines denote the best fit curves to the Randles circuit shown in (**a**).

In order to investigate charge carrier recombination, EIS experiments were performed under visible and UV–Vis light as well in the dark for the P25/Mo-BiVO_4_ bilayer compared to the individual layers (Figure 7). The Nyquist plots were fitted to a Randles equivalent circuit consisting of a series resistance R_S_, the charge transfer resistance R_CT_ at the electrode/electrolyte interface, and a constant phase element CPE (Table 1). Under light irradiation, the films presented lower R_CT_ compared to dark conditions due to the generation of electron-hole pairs. Visible light irradiation resulted in markedly reduced R_CT_ values for the Mo-BiVO_4_ and P25/Mo-BiVO_4_ bilayer in comparison to the P25 films.

This variation can be associated with the weak visible light absorption of the mixed-titania phase of P25 nanocatalysts consisting of (10–20%) rutile nanocrystals with a band gap of ~3.0 eV besides the major (80–90%) anatase phase with smaller (20 nm) nanoparticles and a larger band gap of 3.2 eV [54]. Nevertheless, the charge transfer resistance for the single P25 layer was greatly reduced under UV–Vis light in comparison to the Mo-BiVO_4_ and P25/Mo-BiVO_4_ bilayer, indicating that under these illumination conditions at open-circuit, charge separation and transport are not major obstacles in the PEC performance. Moreover, the R_CT_ values of the P25/Mo-BiVO_4_ bilayer do not vary much with respect to the illumination conditions and the single Mo-BiVO_4_ layer corroborating the proposed charge transfer scheme.

### 3.3. Photocatalytic Performance

**Figure 8:**
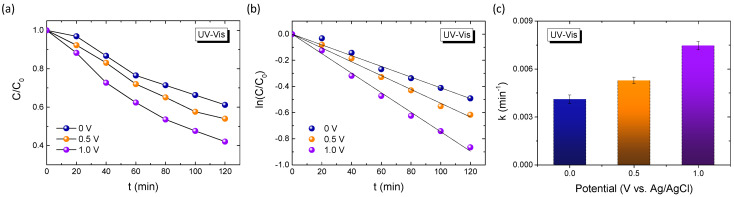
(**a**,**b**) IBU photoelectrocatalytic degradation kinetics and (**c**) corresponding kinetic constants for the P25 films in 0.1 M NaHCO_3_ supporting electrolyte at bias potentials of 0, +0.5, and +1.0 V vs. Ag/AgCl under UV–Vis illumination.

Advanced application of the P25/Mo-BiVO_4_ bilayer films was performed on the photoelectrocatalytic degradation of the pharmaceutical emerging contaminant ibuprofen (IBU), a non-steroidal anti-inflammatory drug that is widely used as an analgesic and antipyretic [61]. It is the third consumable drug and highly detected in many water bodies [62]. It has been shown that IBU is selectively metabolized in humans and animals and the excretion, which is more toxic than the parent molecule, enters into rivers, lakes, oceans, ground water, etc. Initial photocatalytic tests were performed on IBU degradation in 0.1 M NaHCO_3_ supporting electrolyte using the bare P25 films at zero bias potential, +0.5 and +1.0 V vs. Ag/AgCl under UV–Vis light (Figure 8). Dark adsorption of IBU on the P25 films was about 5%, while UV–Vis illumination resulted in the continuous decrease in the pollutant concentration (*C*) with time (Figure 8a), which was detected spectrophotometrically by monitoring the main aromatic absorbance band of IBU at 222 nm [63]. The ln(*C*/*C*_0_) vs. *t* plots, where *C*_0_ is the IBU concentration after dark adsorption, varied linearly with time, indicative of pseudo first-order kinetics (Figure 8b). The values of the kinetic constants *k* obtained from the corresponding linear slopes increased significantly with the applied potential (Figure 8b) that promoted electron-hole separation and pollutant degradation.

**Figure 9:**
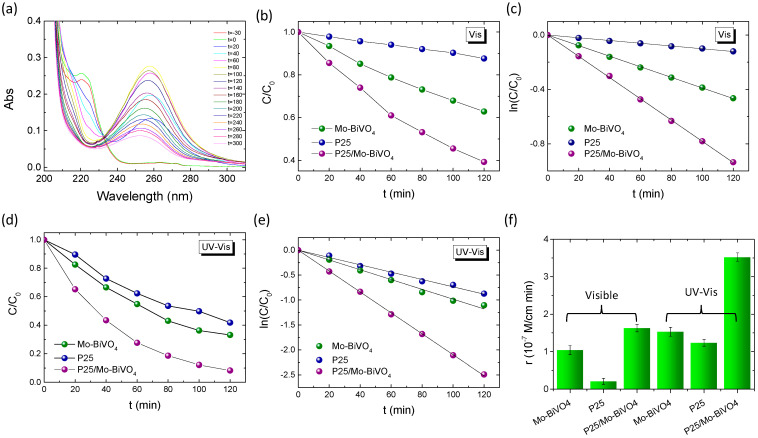
(**a**) IBU absorbance spectra under UV–Vis illumination in the presence of P25/Mo-BiVO_4_. IBU photodegradation kinetics under (**b**,**c**) visible and (**d**,**e**) UV–Vis light as well as the corresponding (**f**) reaction rates for the individual constituents and the P25/Mo-BiVO_4_ bilayer photoelectrodes at +1.0 V vs. Ag/AgCl.

IBU photodegradation was accordingly performed in 0.1 M NaHCO_3_ supporting electrolyte at applied potential +1.0 V vs. Ag/AgCl under visible and UV–Vis light. The reaction kinetics monitored by the corresponding IBU absorbance spectra (Figure 9a) followed pseudo first-order kinetics under both illumination conditions (Figure 9b–e). The photocatalytic reaction rates *r*_Vis_ and *r*_UV-Vis_ were calculated as *r* = *kC*_0_, independent of differences in the initial concentration *C*_0_ after dark adsorption. The degradation of IBU was accompanied by the appearance of a new red-shifted absorption band at about 257 nm, indicative of the formation of reaction intermediates, the most prominent being 4-isobutylacetophenone [64]. The corresponding band reached maximum intensity after 80 min of photocatalytic treatment under UV–Vis light (Figure 9a), while illumination for longer times resulted in its continuous reduction signaling the side group oxidation and oxidative opening of the IBU benzene ring towards mineralization [63,64]. The P25 films showed rather weak reactivity due to the inherently low visible light absorbance of both anatase and rutile TiO_2_ polymorphic phases [65], which increased considerably under UV–Vis light causing electron-hole excitation in P25 titania. The pristine Mo-BiVO_4_ photoelectrodes showed smaller differences in their photocatalytic activity under visible and UV–Vis light, reflecting their intrinsic visible light activation below 490 nm. More importantly, the P25/Mo-BiVO_4_ bilayers presented the highest performance that exceeded the sum of the reaction rate for the individual P25 and Mo-BiVO_4_ layers by approximately 30 and 28% under visible and UV–Vis light, respectively.

**Figure 10:**
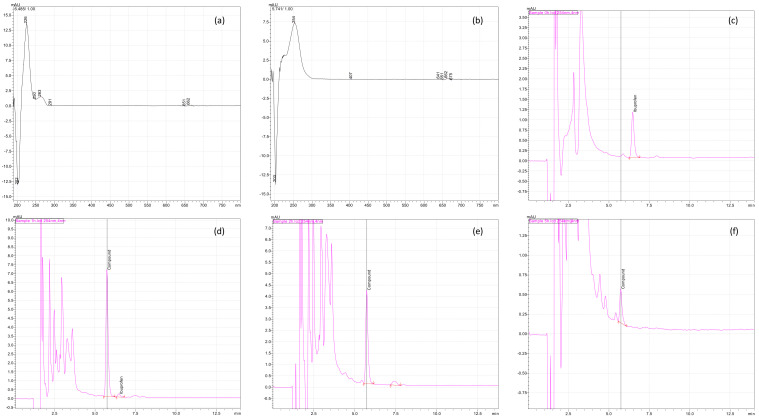
Absorbance spectra of (**a**) IBU and (**b**) the degradation product. HPLC chromatograms of IBU in the presence of P25/Mo-BiVO_4_ films under UV–Vis light for (**c**) 0, (**d**) 1, (**e**) 2, and (**f**) 5 h.

In order to validate the bilayer performance, IBU photodegradation was also monitored by HPLC for the P25/Mo-BiVO_4_ bilayer photoelectrode under UV–Vis light for 5 h (Figure 10). The total run was 15 min and the retention time was 6.3 min for IBU. At 5.4 min, a degradation product of IBU was detected after 1 h, which according to the corresponding UV–Vis spectra (Figure 10a,b) may arise from 4-isobutylacetophenone [64].

The obtained results showed that IBU was completely removed after 2 h (Figure 10e), whereas the degradation of the intermediate reaction product that appeared after 1 h (Figure 10d) reached 94% after 5 h (Figure 10f) under UV–Vis light, supporting the high photocatalytic activity of the bilayer films. Further analytical studies by mass spectrometry techniques would be required, though, in order to identify the transformation products and the reaction pathways during IBU degradation and mineralization [64,66]. Performance comparison with literature results on the photoelectrocatalytic IBU degradation shows that the obtained kinetic constants for the P25/Mo-BiVO_4_ films are comparable to those reported by highly efficient, optimized metal oxide photoanodes (Table 2).

**Figure 11:**
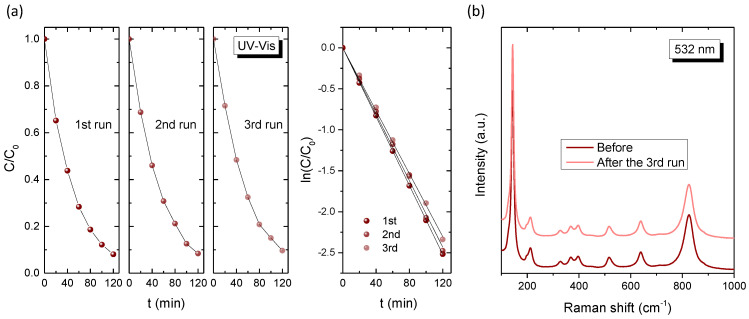
(**a**) Three consecutive IBU photoelectrocatalytic degradation tests using the same P25/Mo-BiVO_4_ bilayer films under UV–Vis light at +1.0 V vs. Ag/AgCl. (**b**) Raman spectra of the bilayer film before and after the third photocatalytic run at 532 nm.

The stability of the P25/Mo-BiVO_4_ bilayer photocatalyst was evaluated by performing three consecutive IBU photoelectrocatalytic degradation tests under UV–Vis light using the same film. The film was subjected to intermediate photocatalytic cleaning of pollutant residues under UV–Vis illumination for 3 h in DI water (Figure 11a). The derived kinetic constants decreased successively from 0.0210(2), to 0.0202(2) and 0.0193(2) min^−1^, after the 2nd and 3rd run, respectively, indicating substantial stability and resistance to photocorrosion given the lack of any co-catalyst or surface treatment of the bilayer photoanode [25]. This was corroborated by Raman measurements of the bilayer film before and after the 3rd photocatalytic test, which showed no sign of film degradation (Figure 11b). Further application and validation of the P25/Mo-BiVO_4_ bilayer photoanodes can be accordingly envisaged in the photoelectrocatalytic degradation of other recalcitrant nonsteroidal anti-inflammatory drugs for water purification, which can be effectively combined with simultaneous hydrogen generation at the cathode under solar light.

## 4. Conclusions

Advanced bilayer photoelectrodes were fabricated utilizing the highly performing Mo-BiVO_4_ PC films as a supporting layer that absorbs visible light below 490 nm, while providing improved charge transport by means of Mo-doping. Spin coating of a mesoporous P25 titania overlayer resulted in a porous heterostructured P25/Mo-BiVO_4_ bilayer structure produced from the densification of the inverse opal underlayer after the second thermal annealing. The bilayer film performance was evaluated on the photoelectrocatalytic degradation of the IBU anti-inflammatory drug, a highly refractory water pollutant of emerging concern, under back-side illumination by visible and UV–Vis light. Significantly enhanced photocatalytic performance was achieved for the bilayer structure with respect to the additive effect of the constituent layers, which, according to optical and PEC measurements, can be associated with the improved light harvesting due to backscattering from the P25 TiO_2_ layer in combination with the favorable interfacial charge transfer at the TiO_2_/Mo-BiVO_4_ heterojunction.

## Figures and Tables

**Table 1:** Parameters obtained by fitting the experimental impedance spectra to a modified Randles circuit for the P25/Mo-BiVO_4_ bilayer in comparison to Mo-BiVO_4_ and P25 films.

	Dark		Visible Light (>400 nm)		UV–Vis Light	
Film	R_S_ (Ω)	R_CT_ (Ω)	R_S_ (Ω)	R_CT_ (Ω)	R_S_ (Ω)	R_CT_ (Ω)
Mo-BiVO_4_	11.9	2.23 × 10^5^	13.1	7292	12.1	4974
P25	9.0	4.62 × 10^5^	20.6	1 × 10^5^	9.1	1909
P25/Mo-BiVO_4_	4.8	26,034	9.4	11,498	9.4	6979

**Table 2:** Performance comparison on IBU photoelectrocatalytic degradation by highly efficient metal oxide photoanodes.

Photoanode	Reactor Cell	Light Source	IBU Concentration	% Degradation	Kinetic Constant	Ref.
BiOI-TiO_2_ nanotube arrays	Double compartment cell, three electrode configuration, 1.2 V vs. Ag/AgCl	100 W Hg lamp	5 mg/L	100% in 2 h	0.0321 min^−1^	[67]
g-C_3_N_4_/Ag/AgCl/BiVO_4_	Single compartment cell, three electrode configuration, 1.23 V vs. RHE, sewage water	150 W solar simulator	2 ppm	–	4.33% h^−1^ cm^−2^	[26]
WO_3_/BiVO_4_	Double compartment cell, three electrode configuration, 1.75 V vs. RHE, 1 M NaCl	Solar simulator 350–1800 nm	100 mg/L	96.7% in 2.5 h	–	[25]
(Ag, Au)/Mo-BiVO_4_	Single compartment cell, three electrode configuration, 1.0 V vs. Ag/AgCl, 0.1 M NaHCO_3_	300 W Xe lamp >400 nm 100 mW/cm^2^	7 mg/L	85% in 4 h	0.0112 min^−1^	[43]
P25/Mo-BiVO_4_	Single compartment cell, three electrode configuration, 1.0 V vs. Ag/AgCl, 0.1 M NaHCO_3_	300 W Xe lamp 100 mW/cm^2^	5 mg/L	100% in 2 h	0.0212 min^−1^	This work

## Data Availability

The original contributions presented in this study are included in the article. Further inquiries can be directed to the corresponding author.
